# Molecular adaptation of a plant-bacterium outer membrane protease towards plague virulence factor Pla

**DOI:** 10.1186/1471-2148-11-43

**Published:** 2011-02-11

**Authors:** Johanna Haiko, Liisa Laakkonen, Benita Westerlund-Wikström, Timo K Korhonen

**Affiliations:** 1 Division of General Microbiology, Department of Biosciences, P.O. Box 56, FI 00014 University of Helsinki, Finland; 2 Neuroscience Center, P.O. Box 56, FI 00014 University of Helsinki, Finland

## Abstract

**Background:**

Omptins are a family of outer membrane proteases that have spread by horizontal gene transfer in Gram-negative bacteria that infect vertebrates or plants. Despite structural similarity, the molecular functions of omptins differ in a manner that reflects the life style of their host bacteria. To simulate the molecular adaptation of omptins, we applied site-specific mutagenesis to make Epo of the plant pathogenic *Erwinia pyrifoliae *exhibit virulence-associated functions of its close homolog, the plasminogen activator Pla of *Yersinia pestis*. We addressed three virulence-associated functions exhibited by Pla, i.e., proteolytic activation of plasminogen, proteolytic degradation of serine protease inhibitors, and invasion into human cells.

**Results:**

Pla and Epo expressed in *Escherichia coli *are both functional endopeptidases and cleave human serine protease inhibitors, but Epo failed to activate plasminogen and to mediate invasion into a human endothelial-like cell line. Swapping of ten amino acid residues at two surface loops of Pla and Epo introduced plasminogen activation capacity in Epo and inactivated the function in Pla. We also compared the structure of Pla and the modeled structure of Epo to analyze the structural variations that could rationalize the different proteolytic activities. Epo-expressing bacteria managed to invade human cells only after all extramembranous residues that differ between Pla and Epo and the first transmembrane β-strand had been changed.

**Conclusions:**

We describe molecular adaptation of a protease from an environmental setting towards a virulence factor detrimental for humans. Our results stress the evolvability of bacterial β-barrel surface structures and the environment as a source of progenitor virulence molecules of human pathogens.

## Background

Analyses of genomic sequences of bacterial pathogens have given an unprecedented view into their biology and evolutionary processes [1,2]. A conclusion from these studies is that highly similar genes, many of which are associated with bacterial virulence, are found across great phylogenetic distances and in different genetic elements, which is indicative of horizontal gene transfer. These families of virulence factors - including toxins, transport systems, adhesins, and antibiotic resistance factors - have evolved by adaptive radiation of a functional progenitor molecule to and within other strains and species to support survival in differing ecological niches [2]. The adaptation, or "evolutionary fine-tuning" of virulence factors that results in increased fitness, can involve modification of catalytic efficiency or substrate specificity of an enzyme, or alteration of bacterial interactions with target cells [2]. The mechanisms of horizontal gene transfer and the functional diversity of bacterial toxin families and protein transport systems have been documented [2-4] but adaptive molecular evolution of bacterial virulence factors remains less understood in terms of altered structure/function relationships.

*Yersinia pestis *is the causative agent of plague, a zoonotic disease transmitted to humans usually by the bite of an infected flea [5]. The bacterium spreads from the intradermal infection site into lymph nodes, causing bubonic plague, and subsequently to blood and to lungs, leading to pneumonic plague. The abilities to disseminate in the host and to cause high bacteremia are central for the transmission of the bacterium by the flea vector which feeds on contaminated blood. *Y. pestis *has been responsible for three human pandemics, which are estimated to have resulted in deaths of ca. 200 million humans [5]. As a bacterial species *Y. pestis *is young, and recent population genetic studies have shown that the bacterium diverged from its ancestral species, the gastrointestinal pathogen *Yersinia pseudotuberculosis*, only ca. 13 000 years ago [6,7]. The genome of *Y. pestis *has evolved through gene decay, recombination, single nucleotide changes, genome rearrangements, and horizontal gene transfer by acquisition of two *Y. pestis*-specific plasmids, of which the plasmid pPCP1 (pPst/pPla) potentiates bacterial dissemination from the primary intradermal infection site into lymph nodes [8,9]. The decisive virulence factor encoded by pPCP1 is the surface protease plasminogen activator Pla. Deletion of *pla *attenuates *Y. pestis *millionfold in subcutaneously infected mice, whereas no difference is seen in intravenously infected mice [8]. Pla is specifically needed for establishment of bubonic plague [10,11], and a critical role of Pla has been described in pneumonic plague where it enables localized growth of *Y. pestis *in the lungs [12].

Pla belongs to an outer membrane protease family of omptins that have been detected in several Gram-negative bacteria of different phylogenetic groups; these bacteria commonly infect animals or plants [13]. The omptin genes have spread through horizontal gene transfer by different mechanisms, and at least 16 members are known to date [13-16]. As omptin sequences are over 50% identical, they very likely fold similarly to the two structurally resolved members of the family, OmpT of *E. coli *[17] and Pla of *Y. pestis *[18]. Both OmpT and Pla form a 70-Å long β-barrel of elliptical cross-section with ten antiparallel transmembrane β-strands, five surface-exposed loops (L1-L5) and four short periplasmic turns (T1-T4). The catalytic residues and the acidic, substrate-binding pocket, as well as the lipopolysaccharide-binding site are conserved in omptins [13,17,19]. This is in accordance with the finding that the cleavage site preferences of omptin proteolysis, analyzed mostly with short peptide substrates, are very similar: omptins cleave after basic residues, preferably between two basic amino acids [20-23]. With larger substrates, however, omptins are functionally diverse, and their genes have undergone adaptive evolution to support the life style of the bacterial host [14,15,24]. Insertions and deletions that give slight variation in the molecular sizes of omptins are mostly located in the solvent-accessible surface structures L1-L5, and the differential substrate selectivities of individual omptin proteases appear to be dictated by these surface loops, as observed by loop swapping and omptin chimeras [16,19,25,26].

During infection, Pla seems to increase virulence mainly by interfering with the human plasminogen/fibrinolytic system that is critical in cell migration across tissue barriers. Pla cleaves the circulating, abundant zymogen plasminogen by a single cut to active plasmin [8] and also degrades two natural inhibitors of the plasminogen system, the serpins α_2_-antiplasmin (α_2_AP) and plasminogen activator inhibitor 1 (PAI-1) [16,19]. Altogether, these activities result in uncontrolled plasmin activity. Plasmin is a broad-substrate protease involved in a number of (patho)physiological processes, of which fibrinolysis and damage of extracellular matrices contribute to bacterial dissemination within the host [12,27,28]. Pla has also non-proteolytic functions: it mediates invasion of *Y. pestis *into human epithelial and endothelial cells by an unknown mechanism as well as into mouse monocytes by binding to CD205 [29-31]. The Epo omptin is a close homolog of Pla, exhibiting 77% sequence identity, and it is encoded on a plasmid of the plant pathogen *Erwinia pyrifoliae *that causes tissue-destructive infection in pear trees [32]. The functions of Epo in plant pathogenesis have not been systematically studied, but serpinolytic activity *in vitro *was recently shown [16].

The claim of directed evolution and adaptive molecular evolution is that natural selection generates particularly evolvable enzymes in response to rapidly fluctuating selective conditions and that proteins that require fewest mutations to adapt to novel conditions are the most likely to survive environmental changes [33]. Evolvability has been argued to be a function of robustness, i.e., the capacity of a protein to withstand sequence variations without disruption of its binding or catalytic properties [33,34]. Mutational robustness is dependent on a protein's intramolecular interactions, and the omptin molecule fulfils the criteria of an evolvable protein as defined by O'Loughlin *et al. *[33]; the β-barrel is a sturdy membrane-embedded molecule with flexible surface loops that can tolerate deletions and insertions without compromising molecular stability [35,36]. Manifestations of the robustness of omptins include their unhurt catalytic activity after autocatalytic cleavage at L4 and the proteolytic specificity of omptin loop chimeras, which together have indicated that the five loops offer a flexible scaffold for recognition of different substrates [16,19,25,26].

Evolutional studies on bacterial virulence factors highlight the importance of environment as an immense reservoir of evolvable potential progenitors of virulence factors [2]. Pla of *Y. pestis *and Epo of *E. pyrifoliae *belong to the same subfamily of omptins which also contains the omptins PgtE of *Salmonella enterica*, Kop of *Klebsiella pneumoniae*, and the omptin of *Enterobacter *[15,16]. These bacterial species infect mammals or plants or both, and have encountered mutual horizontal gene transfer. In this study we used the existing knowledge of omptin structure and function as a starting point for substitutional analysis, and addressed two specific questions on molecular adaptation: how many substitutions are needed and at which locations in Epo to gain a proteolytic function (plasminogen activation) and a non-proteolytic function (invasiveness) expressed by Pla.

## Results

### Substrate selectivity of Pla and Epo towards virulence-associated human proteins

**Figure 1:**
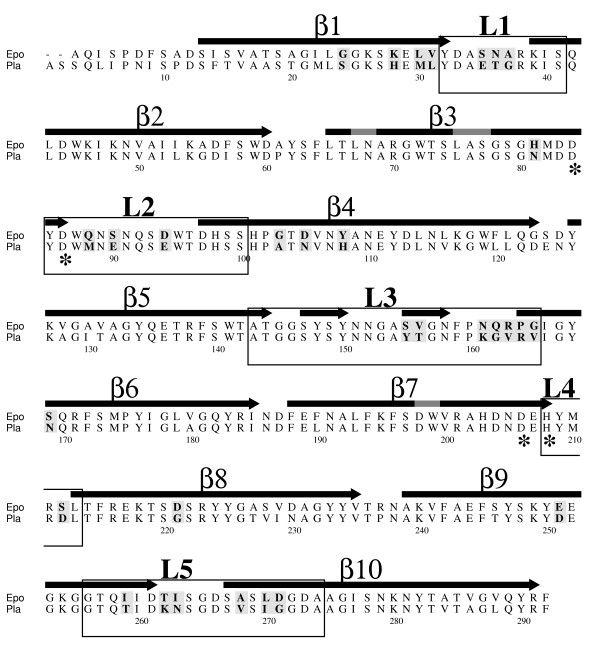
**Sequence alignment of Epo and Pla**. The β-strands and the extracellular loops are numbered, and the 31 differing amino acids in the extracellular side of the protein are shown in bold face in gray background. The catalytic residues are indicated with asterisks. The sequences were aligned with ClustalW [50], and the image was rendered with Alscript [51]. The secondary structures are defined by Stride [52], and they are based on the known Pla structure [18].

We began this study by comparing the activity of Pla and Epo in Pla-mediated functions that i) have been documented to be important in plague pathogenesis *in vivo*, such as plasminogen activation by cleavage, or ii) directly or indirectly enhance the virulence potential *in vitro*, such as degradation of α_2_AP and PAI-1 and invasion to the human endothelial-like cell line. Mature Pla sequence differs from Epo by 65 amino acids, of which 31 are in protein regions located outward the outer membrane lipid bilayer. Of these 31 differences, 18 are located in the surface loops L1-L5 (Figure 1). For these assays, we expressed *pla *and *epo *in a well-defined laboratory host strain *E. coli *XL1 to avoid background problems with other virulence characteristics of *Y. pestis *and *E. pyrifoliae*, which either have been shown to or might interfere with the proteolytic activity of Pla or Epo [37-39].

**Figure 2:**
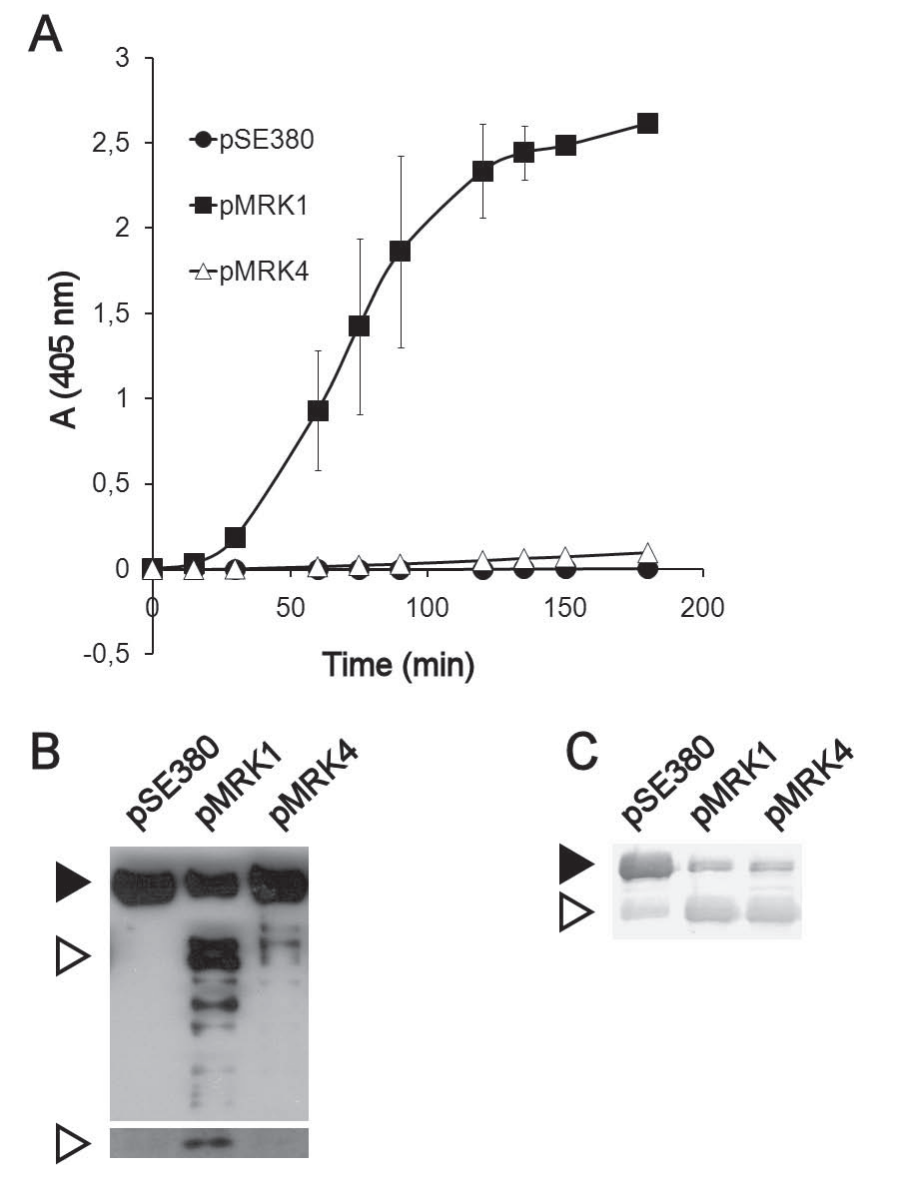
**Comparison of Pla and Epo**. Pla and Epo were compared in (A) plasminogen activation, (B) degradation of plasminogen, and (C) degradation of α_2_-antiplasmin. *pla *and *epo *were expressed in recombinant *E. coli *XL1. The strains are indicated with their plasmid names (see Table 1). In (A), plasmin formation was measured using a chromogenic plasmin substrate. The data are average of two independent assays with duplicate samples, and standard deviations are shown. In (B), Western blotting with anti-plasminogen (top panel) and anti-plasminogen catalytic domain (bottom panel) antibodies is shown. The black arrowhead indicates plasminogen and the white arrowheads indicate cleaved plasminogen, the heavy chain (top panel) and the light chain (bottom panel) of plasmin. In (C), degradation of α_2_AP was visualized with Western blotting using α_2_AP antibody, and the black arrowhead indicates uncleaved α_2_AP and the white arrowhead indicates the degradation product.

A clear functional difference between Pla and Epo was observed in plasminogen activation: Pla activated plasminogen efficiently but Epo very poorly during a 3-h cumulative measurement (Figure 2A). We next analyzed plasminogen degradation by Pla and Epo using Western blotting with two different antibodies: i) polyclonal anti-human plasminogen antibody that mainly detects the charged kringle domains in the heavy chain of plasmin, and ii) monoclonal anti-human plasminogen antibody that recognizes the catalytic domain in the light chain of plasmin (Figure 2B). Epo expressed by *E. coli *XL1 (pMRK4) degraded plasminogen but significantly more slowly than did Pla expressed in the same host strain in a 2-h incubation with plasminogen (Figure 2B). Further, Epo did not form detectable light chain of plasmin in a 3-h incubation with plasminogen, which however was discernible with the Pla-expressing bacteria (Figure 2B). We have recently reported that Epo and Pla degrade the serpin PAI-1 [16], and recombinant *E. coli *with Pla or Epo also cleaved another serpin, α_2_AP (Figure 2C). Thus, both omptins exhibited similar serpinolytic activity but differed in plasmin formation.

### Construction of Pla-Epo hybrid proteins

**Figure 3:**
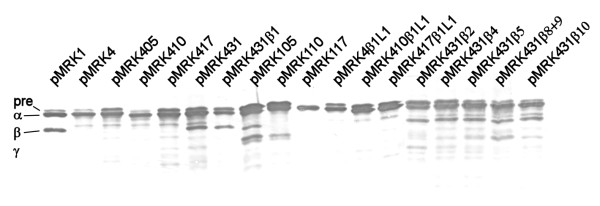
**Analysis of expression of Pla-Epo hybrid proteins in *E. coli***. Expression of the Pla-Epo-hybrid proteins was analyzed by Western blotting of whole cells with a mixture of anti-Pla, anti-Epo, and anti-Pla-loop antisera. The constructs are indicated with their plasmid names (see Table 1). Migration distance of the Pla isoforms are indicated; pre-Pla is the immature form of Pla, α-Pla and γ-Pla are mature Pla proteins with differing conformations, and β-Pla is the mature autocleaved form of Pla.

In order to define the structural differences between Pla and Epo that govern the Pla-like plasminogen activation, we analyzed the sequence differences of Pla and Epo, especially at the surface accessible regions which are important for polypeptide substrate selectivity of omptins (see Figure 1) [19,25]. We generated Pla-Epo hybrid proteins by swapping amino acid residues in the loop regions differing between Pla and Epo sequences, and then expressed the proteins in *E. coli *XL1. We initially selected the residues to be substituted on the basis of their clustering and location at the loops or on the extracellular side on the protein. Our approach was to create cumulative substitutions, first at those loop regions where the most striking amino acid differences between Pla and Epo are, and then at the amino acids located on the extracellular side of the protein above the lipid bilayer. Initially, the substitutions and analyses were made in two directions: positive alteration from Epo towards Pla (i.e., gain of function) and negative alteration from Pla towards Epo (i.e., loss of function). The nomenclature of the hybrid-expressing plasmids is as follows: the first number tells the origin (1 for Pla, 4 for Epo), the next two numbers tell the number of substitutions made in the mature protein, and the possible change of β strands is then given (see Table 1). E.g., pMRK431 denotes the Epo-based hybrid with 31 amino acid substitutions towards Pla and pMRK431β2 a hybrid additionally substituted for the β2 strand. The hybrid proteins were expressed from the same high-copy plasmid in the same host strain; however, we also analyzed their expression by Western blotting of whole cells and of cell wall preparations using a cocktail of anti-Pla, anti-Epo, and anti-Pla loop-specific sera (Figure 3). These assays did not reveal substantial differences in the protein expression levels that would explain, according to our previous experience [19,37,39], the observed differences in plasminogen activation. The assay however is semi-quantitative as the different hybrid proteins vary in reactivity with the antibodies.

**Table 1:** Bacterial strains and plasmids used in this study

Bacterial strain or plasmid construct	Description	Reference
*Escherichia coli *XL1 Blue MRF'	Δ(*mcrA*) 183 Δ(*mcrCB-hsdSMR-mrr*) 173 *endA1 supe44 thi-1 recA1 gyrA96 relA1 lac *[F' *proAB lacI*^*q*^*ZΔ M15 *Tn *10 *(tet)]	Stratagene

pSE380	Expression vector, *trc *promoter, *lacO *operator, *lacI, bla*	Invitrogen

pMRK1	*pla *in pSE380	[19]

pMRK4	*epo *in pSE380	[16]

pMRK105	Pla with 5 aa from Epo: ^**161**^**KGVRV**→**NQRPG**	This study

pMRK405	Epo with 5 aa from Pla: ^**159**^**NQRPG**→**KGVRV**	This study

pMRK110	Pla with 10 aa from Epo: ^161^KGVRV→NQRPG, ^**262**^**KN**→**TI**, ^**268**^**VSIG**→**ASLD**	This study

pMRK410	Epo with 10 aa from Pla: ^159^NQRPG→KGVRV, ^**260**^**TI**→**KN**, ^**266**^**ASLD**→**VSIG**	This study

pMRK117	Pla with 17 aa from Epo: ^**35**^**ETG**→**SNA**, ^**88**^**MNE**→**QNS**, ^**155**^**YT**→**SV**, ^161^KGVRV→NQRPG, ^262^KN→TI, ^268^VSIG→ASLD	This study

pMRK417	Epo with 17 aa from Pla: ^**33**^**SNA**→**ETG**, ^**86**^**QNS**→**MNE**, ^**153**^**SV**→**YT**, ^159^NQRPG→KGVRV, ^260^TI→KN, ^266^ASLD→VSIG	This study

pMRK431	Epo with 31 aa from Pla: ^33^SNA→ETG, ^86^QNS→MNE, ^153^SV→YT, ^159^NQRPG→KGVRV, ^260^TI→KN, ^266^ASLD→VSIG, ^**26**^**KELV**→**HEML**, ^**101**^**GTDVNY**→**ATNVNH, I257T, H79N, D92E, S210D, S167N, D219G, E249D, G22S**	This study

pMRK431β1	Epo with 42 aa from Pla + the signal sequence: N-terminus from Pla until aa 45; the rest similar to pMRK431	This study

pMRK4β1L1	Epo with the N-terminus from Pla until aa 45	This study

pMRK410β1L1	pMRK410 with the N-terminus from Pla until aa 45	This study

pMRK417β1L1	pMRK417 with the N-terminus from Pla until aa 45	This study

pMRK431β2	pMRK431 with β2 from Pla	This study

pMRK431β4	pMRK431 with β4 from Pla	This study

pMRK431β5	pMRK431 with β5 from Pla	This study

pMRK431β8+9	pMRK431 with β8 & β9 from Pla	This study

pMRK431β10	pMRK431 with β10 from Pla	This study

Bold text indicates the novel substitutions compared to previous hybrid. aa, amino acid

### Plasminogen activation by Pla-Epo hybrid proteins

**Figure 4:**
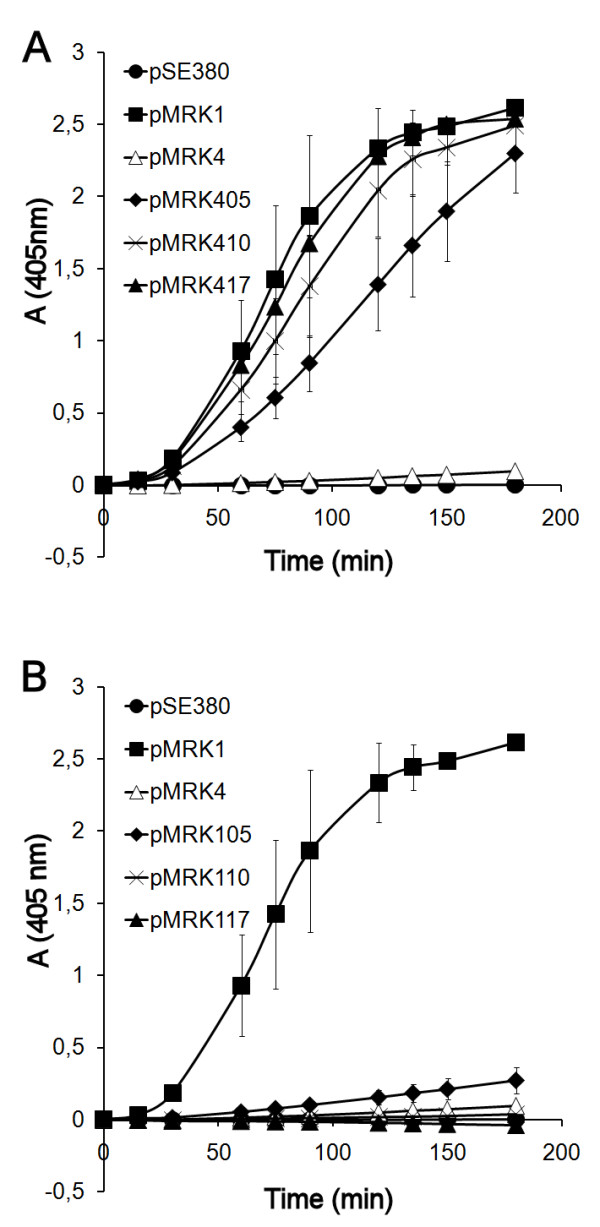
**Cumulative plasminogen activation by Pla-Epo hybrid proteins expressed in *E. coli***. (A) Pla-Epo hybrids with cumulative substitutions towards Pla. (B) Pla-Epo hybrids with cumulative substitutions towards Epo. Inserts show the plasmid constructs (see Table 1). The data are average from two independent assays with duplicate samples, and standard deviations are shown.

Cumulative plasminogen activation assays [19], where the recombinant *E. coli *were incubated with plasminogen and the chromogenic plasmin substrate, were performed with Pla-Epo chimeras. The plasmid pMRK405 encodes the substitution of the loop 3 sequence ^159^NQRPG of Epo to the sequence ^161^KGVRV of Pla (see Figure 1 and Table 1), and this substitution in Epo resulted in efficient plasminogen activation (Figure 4A). The reverse substitution in *pla*, encoded in plasmid pMRK105, nearly completely abolished plasminogen activation by recombinant *E. coli *(Figure 4B). We next analyzed the effect of the further substitutions ^260^TI/KN and ^266^ASLD/VSIG which are located at L5 in Epo and encoded in pMRK410, and this hybrid protein was nearly as effective as Pla in plasminogen activation (Figure 4A). The corresponding substitutions in Pla encoded in plasmid pMRK110 gave a protein with very low plasminogen activation (Figure 4B). Further loop substitutions in Epo-based plasmid pMRK417, i.e. ^33^SNA/ETG at L1, ^86^QNS/MNE at L2 and ^153^SV/YT at L3, only slightly improved plasminogen activation. The reverse substitutions in pMRK117 gave a hybrid protein that did not activate plasminogen at all. Thus, the substitution of 10 out of the 290 amino acids in the mature Epo protein, located at L3 and L5, was enough to significantly alter its protease substrate selectivity towards activation of human plasminogen.

The stepwise accumulation of substitutions onto the Epo molecule was continued onwards from pMRK417 to pMRK431, where all 31 residues that differ between Epo and Pla and that are located on the extracellular side of the outer membrane on the protein have been substituted towards Pla. These substitutions in Epo did not change the cumulative plasminogen activation or the fragmentation pattern of plasminogen (data not shown). We recently reported that the single substitution I259T at loop 5 differentiates Pla of the pandemic *Y. pestis *lineages (which have PlaT259) from the Pla isoform of the ancestral lineages (PlaI259). This substitution decreases Pla-mediated cleavage of plasmin light chain that contains the catalytic domain, and thus increases the stability of the formed plasmin [26]. An increase in plasmin stability and formation of the plasmin light chain after the I259T substitution was also observed in the series of Pla-Epo hybrid proteins in the present study.

### Structural analysis

**Figure 5:**
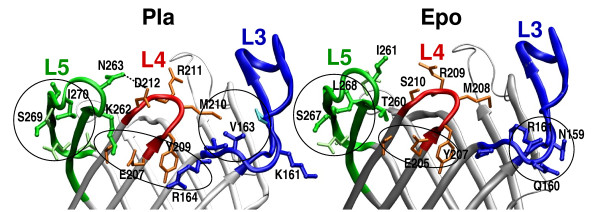
**Analysis of the ten substituted residues in Pla structure (left) and Epo model (right)**. L3 and its residues are colored blue, L4 red, and L5 green. The substituted residues are drawn thick and in a darker shade, and the other discussed residues thin and in lighter shade. The hydrogen bond between D212 and N263 in Pla is marked with a dashed line, and three interaction areas circled in both structures. Counting from left: a tight cluster of hydrophobic residues in L5; a polar quintet between L5, L4 and L3 in Pla and a L4 triplet in Epo; hydrophobic contacts within the barrel opening in Pla and polar residues at the outside of the barrel in Epo.

To gain physical insight into the observed functional differences in proteolysis, we studied the roles of the swapped residues in the crystal structure of Pla [18] and the homology model of Epo. The immediate environment of the residues substituted in pMRKX10, where X is either 1 or 4, is shown in Table 2 and Figure 5. The first five substituted amino acids occur at the very end of the long loop L3 and the following five are found at the tip of L5 and in the early, solvent-accessible part of strand β10. There are no direct contacts between ^159^NQRPG/KGVRV and ^260^TI/KN or ^266^ASLD/VSIG, but both the L3 and the L5 residues interact with the intervening L4. The β7-strand before L4 contains active site residues D206 and H208, and is fully conserved between Epo and Pla except for one residue (S210/D212; see Figure 1).

**Table 2:** Molecular environment of the substituted sites in Pla structure and Epo model

	Pla	Contact	Epo	Contact
**MRKX05**	**K161**	solvent	**N159**	**Q160, R161**

	**G162**	-	**Q160**	**N159, R161**

	**V163**	**p160**, m210	**R161**	**N159, Q160, p158**

	**R164**	y209, t142	**P162**	**R161**

	**V165**	H101, a143, **g145, i166**	**G163**	-

*MRKX10*	*K262*	e207, t214	*T260*	S210, t212, **d259**

	*N263*	D212, *d261*	*I261*	*d259*

	*V268*	*I270*	*A266*	**i257**

	*s269*	*q258*	*s267*	*q256*

	*I270*	*t257, V268, a274*	*L268*	-

	*G271*	-	*D269*	a32, *g270*

Residues whose side chains come within 4 Å of the central amino acid are listed. The amino acids in each loop are indicated in different font styles, **L3 (G145-V165) in bold**, L4 (H208-L213) underlined and *L5 (G255-A274) in italics *in Pla numbering.

Residues that are conserved at the site are shown in lower case letters, and the non-conserved ones in capital letters.

The five residues of the first substitution site are the longest consecutive difference between Pla and Epo. In the Pla structure, V163 at L3 packs together with apolar residues P160 at L3 and M210 at L4 at the inside of the barrel, while the L3 residues N159, Q160, and R161 in Epo point towards the water phase (Figure 5). The distinct functional effect of pMRK405 substitutions could well reflect changes in size of the binding pocket and differences in the electrostatic properties of the substrate docking surface.

The incremental substitutions of pMRK410 occur in L5. K262 and N263 are located in the very tip of L5, and in the Pla structure, both residues are in contact with L4. Most intriguingly, N263 forms a hydrogen bond with Pla-specific D212 in L4. Such an interaction is not possible between the corresponding residues in Epo, S210 and I261. Moreover, K262 of Pla points towards E207 in β7. This glutamate, and the threonine and the tyrosine hydrogen-bonded to it are identical in Pla and Epo, but the polar triplet at the outside of the barrel gets extended and strengthened in Pla by K262 (L5) from one side and by R164 (L3) from the other (Figure 5). The corresponding Epo-residues T260 and P162 are decisively smaller, and do not reach to the L4 residues. pMRK410 construct also includes swapping of ^268^VSIG/^266^ASLD in L5. These residues lie in a small β-structure [18], supposedly in the aqueous phase above the lipopolysaccharide layer. The residues in both proteins form similar interactions with their neighbors in same loop, except the Epo-specific D269 that in the model hydrogen bonds to a backbone carbonyl in the β1-strand. The end of loop L5 and the beginning of L1 are rather irregular; both areas differ between Pla and Epo, and a side chain-backbone hydrogen bond would stabilize the loops relative to each other. In the Pla structure, L1 residues D33-R38 form a network of hydrogen bonds.

### Serpinolytic activity of Pla-Epo hybrids

**Figure 6:**
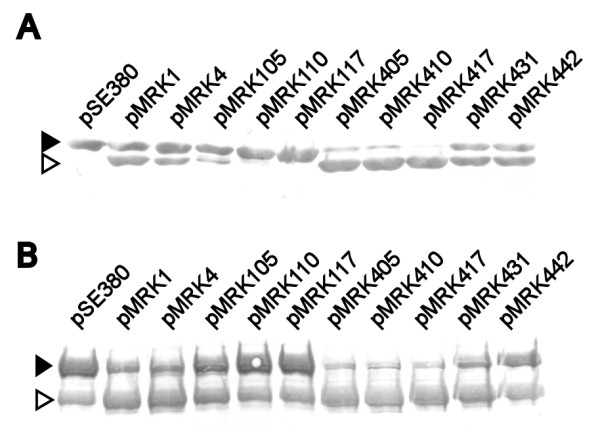
**Degradation of serpins by *E. coli *expressing the Pla-Epo hybrid proteins**. Degradation of PAI-1 (A) and α_2_AP (B) in a 2-h incubation was analyzed by Western blotting. The black arrowhead indicates uncleaved serpin and the white arrowhead indicates the degradation product. The strains are indicated with their plasmid names (see Table 1).

To study the effect of structural changes on degradation of another substrate, we next analyzed the degradation of PAI-1 and α_2_AP by the Pla-Epo chimeras. Pla and Epo degraded PAI-1 and α_2_AP in a similar manner (Figure 6). Recombinant *E. coli *strains (pMRK405), (pMRK410), and (pMRK417) cleaved PAI-1 and α_2_AP more efficiently than did *E. coli *with Pla or Epo (Figure 6). On the other hand, the hybrids with further substitutions, encoded in plasmids pMRK431 and pMRK431β1, caused slower degradation of both PAI-1 and α_2_AP, similar to that seen with Pla and Epo. The Pla-derived hybrid proteins, encoded by plasmids pMRK105, pMRK110, and pMRK117, were severely decreased in their serpinolytic activity compared to both Pla and Epo. Thus, serpinolytic activity and plasminogen activation seem to require different amino acids in the omptins.

### Turning Epo into an invasin

Invasiveness of *Y. pestis *into human epithelial and endothelial cells is a putative virulence-associated property of Pla [29,30], which was detected in a gentamicin protection assay with *E. coli *(pMRK1) expressing Pla but not with *E. coli *(pMRK4) expressing Epo (Table 3). To compare gain of invasiveness to gain of plasminogen activation, we tested the Pla-Epo chimeras for their effect on invasion into the ECV304 cell line. *E. coli *expressing chimeras up to pMRK417 gave a modest increase in bacterial invasiveness. Surprisingly, *E. coli *XL1 (pMRK431) also showed only a low but clearly positive level of invasiveness, indicating that substitution of all extramembranous residues differing between Pla and Epo, sufficient to gain Pla-like plasminogen activation, was not enough to turn Epo into an efficient invasin. Strikingly, further substitutions in the transmembrane region of Epo in pMRK431β1 (expressing all 31 Pla-specific residues located above the membrane and the remaining five residues, F14, T15, A18, T20, and M22, from the N-terminal β1-strand and the irregular region before the β1-strand) induced invasiveness to the level seen with *E. coli *(pMRK1) expressing Pla. The hybrid encoded in pMRK431β1 was similar to pMRK431 in enhancing plasminogen activation by *E. coli *(data not shown). Fusion of the same 5' *pla *DNA (thus including the β1-strand and the L1) into *epo *or the hybrids pMRK410 or pMRK417 did not induce similar invasiveness (Table 3), indicating that the Pla-like omptin surface is needed and the amino terminus alone does not potentiate invasion. Further, substitution of the other β-strands to pMRK431 construct did not influence invasiveness (Table 3). We substituted in pMRK431 the β strands 2, 4, 5, 8 and 9 together, and 10, that vary most in the β-strand sequences of Pla and Epo.

**Table 3:** Results of the invasion assay

*E. coli *XL1	Average invasion percentage	Number of assays	Range
pSE380	0,0	33	0-0.3

pMRK1	4,4**	33	0.7-24.8

pMRK4	0,0	9	0-0.05

pMRK417	0,1*	9	0-0.2

pMRK431	0,5	6	0-1.9

pMRK431β1	5,4**	14	0.4-16.3

pMRK4β1L1	0,0	4	0-0.04

pMRK410β1L1	0,1	6	0.02-0.3

pMRK417β1L1	1,2	6	0.04-5.4

pMRK431β2	0,4	5	0.1-0.8

pMRK431β4	0,4	5	0.02-1.2

pMRK431β5	0,3	5	0.03-0.9

pMRK431β8+9	0,1	5	0.02-0.2

pMRK431β10	0,2*	6	0.06-0.3

Gentamicin protection assay with the human ECV304 cell line. The results are given as the percentage of the intracellular vs. the total added bacteria. The strains have been compared to *E. coli *XL1 (pSE380) to calculate significant differences with t-scores. * p < 0.05, ** p < 0.01

## Discussion

*Erwinia *species and *Y. pestis *colonize different habitats and have a very different pathogenic potential for humans. Bacterial virulence is normally dependent on several factors, and presence of Pla, although it is an exceptionally multifunctional and powerful virulence factor [8,10-12,16,19,26-28,30,31] does not alone explain the devastating virulence of *Y. pestis*. Our study however gives an example of how a single, specific virulence factor of the plague bacterium might have structurally adapted to its present form. Our results do not give a direct roadmap to the Pla molecular structure but rather describe how easily the substrate specificity of an omptin can evolve and how functions that are pathogenic in a novel environment can be gained. The role of surface loops in Pla proteolysis has earlier been documented by loop swapping [16,26], and our results extend these findings by a more detailed substitutional analysis and by also conferring invasiveness onto a close ortholog of Pla.

Serpins are important regulators of cellular proteolysis and share structural features, which include a similar, exposed reactive center loop that binds to and inhibits the catalytic center of target proteases [40]. The omptins in the Pla subfamily all cleave specifically the reactive center loop of the PAI-1 [16]. Here we found that Epo can cleave the serpin α_2_AP, which is a shared target of Pla and PgtE [19,41]. These findings indicate that serpinolytic activity is a common function in the Pla subfamily of omptins and might give selection advantage for survival and spread of bacteria in their hosts by increasing proteolysis in tissues. In contrast, omptins of the OmpT subfamily, OmpT of *E. coli *and SopA of *Shigella*, do not cleave PAI-1, despite high sequence similarity, conserved fold and active site structure [16]. It is interesting to note that the Pla-Epo hybrid proteins encoded in pMRK405, pMRK410, and pMRK417 were more efficient than Pla or Epo in PAI-1 degradation. Also, further substitution towards Pla, encoded in pMRK431 and pMRK431β1, led to hybrid proteins that exhibited an activity similar to that of Pla and Epo. Acquisition of novel substrate specificities by proteases often leads to broad-specificity variants by initially favoring mutations that increase the conformational flexibility and destabilize specialized active site structures. thus reducing specificity for the original targets [33]. The observation that intermediate Pla-Epo hybrid proteins were more efficient in PAI-1 degradation is in line with the assumption that serpinolytic activity is a property of a progenitor omptin in this subfamily and that Pla and Epo have diverged from this progenitor to have novel functions. The structural explanation for the increased efficiency is however unknown. The activating cleavage in plasminogen occurs between the R-V bond, while in PAI-1 and probably also in α_2_AP the cleavage occurs between R-M in the reactive center loop. Specialization to plasminogen cleavage by Pla seems to correlate with several stabilizing interactions between the surface loops, formed by Pla-specific residues.

A molecular explanation for the altered plasminogen activation encoded by pMRK410 remains speculative without more specific molecular data about omptin-target complexes, and more generally, because the *in vivo *mobility of the surface structures of the omptin barrel. Still, the structural analysis presented here suggests a possible explanation. Most importantly, we see multiple loop-loop interactions involving Pla-specific residues. These would result in reduced loop flexibility and a better defined binding pocket, promoting productive substrate binding. Second, we see a binding pocket altered in size and hydrophobicity. The pMRKX05 (where X is either 1 or 4) substitutions ^161 ^KGVRV/NQRPG occur at one long end of the elliptical barrel opening, and the ^268^VSIG/ASLD at the other. This seems to imply that the polypeptide substrates bind across the long axis of the omptin pore, and the pMRKX10 mutations change the docking surface. Altogether, the Pla structure seems to allow close approach by the substrate, and the active site to be well stabilized by interloop interactions. Assuming this is the reason for the observed differences in plasminogen cleavage versus serpinolysis, it means that plasminogen binding and cleavage require a "preformed", tighter binding pocket.

The protein sites identified in this study enable the omptin to initiate activating cleavage of plasminogen but further substitutions are needed to stabilize plasmin. Swapping of loops between Pla and PgtE of *S. enterica *has revealed that L5 and the N-terminal region including L1 are crucial for the gelatinase activity by PgtE [25]. The same regions and in addition L3 seem to be important in the activation of pro-matrix metalloproteinase 9 by PgtE [25]. A residue deserving special attention is T259 of Pla, which is situated in L5 and faces into the barrel opening next to F215. We recently described that a single substitution at this site, T259I that is typical for ancestral *Y. pestis *lineages, increases the cleavage of the light chain of plasmin, thus decreasing the stability of the formed plasmin [26]. The conservation of this mutation in pandemic isolates indicates that *pla*, after being acquired by *Y. pestis*, has evolved through a single nucleotide change to better support plasmin formation. The pPCP1 plasmid also encodes pesticin and pesticin immunity protein and is evolutionary related to colicin plasmids, in particular pColE1[42]. It is possible that the pPCP1 plasmid has evolved through fusion of a *pla *progenitor gene and a ColE1-like plasmid.

The omptins autoprocess themselves at different protein sites [15]. Formation of β-Pla-like peptide by Epo hybrid proteins was detected by *E. coli *expressing pMRK431 that encodes the substitution of all 31 differences between Pla and Epo at their surface-exposed parts. This indicates that autoprocessing is dependent on surface loops. Unexpectedly, these substitutions only slightly improved the invasiveness of recombinant *E. coli*. This indicates that the proteolysis and the invasiveness involve different regions of the omptin molecule and that the invasiveness, which among omptins has been detected only with Pla [15], is a more complex function than the proteolytic specificity. The plasmid pMRK431β1, constructed from pMRK431 to also encode the amino terminal residues 1-45 containing the β1-strand, induced similar level of invasiveness as seen with *E. coli *pMRK1. However, Pla amino terminus alone in Epo did not convert Epo to an invasin. At present we do not have an explanation for the requirement of transmembrane regions to induce invasion by Epo. Our hypothesis was that this might indicate TonB-dependency in invasion. TonB functions in energy coupling between the cytoplasmic and the outer membrane and is important in transport functions across the bacterial cell wall [43]. The Pla and Epo sequences contain a homolog to the TonB box [44] at the amino terminus (^11^DSFTVAA in Pla and ^9^DSISVAT in Epo). A role for TonB in Pla-mediated invasion however seems unlikely, as expression of Pla or Epo in a *tonB*-deletion of *E. coli *XL1 mutant did not alter their invasiveness (T. Lehti and J. Haiko, unpublished). One possibility is that invasion requires membrane fusion which could differ between Epo and Pla, as outer membrane β-barrels are known to vary in their incorporation into different lipid micelles [45]. It is also possible that changes in membrane-embedded residues have subtle effects on the orientation and activity of surface residues that make contact with the target cell. Finally, as a molecular event invasion is complex and may require several steps (adhesion, membrane fusion, host cell responses) that involve more than one omptin protein region. This is in line with the finding that extensive substitution was required to induce invasiveness into Epo.

## Conclusions

**Figure 7:**
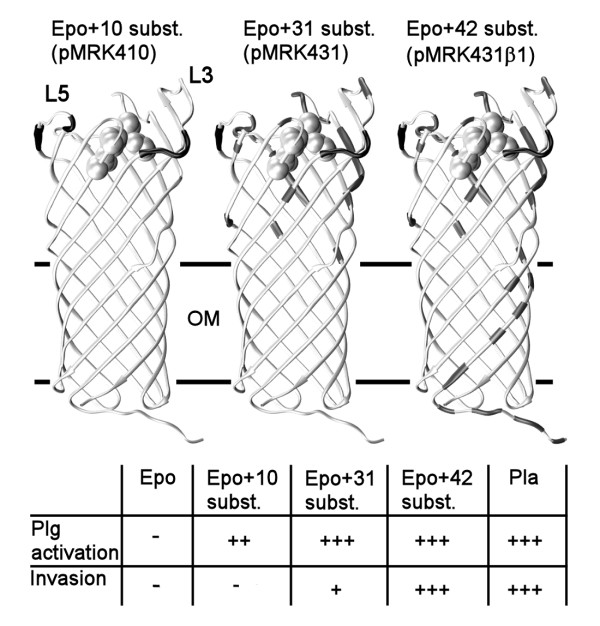
**Summary of the substitution analysis**. Molecular models of selected Pla-Epo hybrid proteins are shown, with the substituted sites coloured black. The outer membrane (OM; the girdle area) and loops 3 and 5 are indicated. The active site amino acids are shown in a space filling representation. The table summarizes the plasminogen (Plg) activation and invasion capacities of Epo, Pla, and the Pla-Epo hybrid proteins with 10, 31, and 42 substitutions towards Pla; in total, there are 65/292 amino acid differences between mature Pla and Epo. For clarity, also the plasmid names are shown.

The omptin β-barrel fold has spread through horizontal gene transfer in Gram-negative bacteria, and their pathogenetic functions and potential vary significantly and correlate well with the pathogenetic mechanisms of the host bacterial species. This implies that the omptins have adapted and contribute to the life style of the bacteria, while they also have retained common functions such as serpinolytic activity. Pla and plasminogen activation are central in plague pathogenesis, and among the omptins, Pla is by far the most efficient plasminogen activator while other omptins either cleave plasminogen poorly or not at all [15,19,26]. Change of a few Epo amino acids to those in Pla conferred a plague virulence function on the omptin from a plant pathogen. The mature sequences of Pla and Epo differ at 65 out of 292/290 residues, and we observed that the substitution ^159^NQRPG/KGVRV at L3 alone caused a substantial improvement in the plasminogen activation ability of Epo when expressed in *E. coli*. Further substitutions ^260^TI/KN and ^266^ASLD/VSIG at L5 improved the initial plasminogen activation close to the level seen with Pla-expressing *E. coli*. Structural analysis indicated that differing residues at L3 and L5 in Pla and Epo interact with L4 in different ways, leading to differences in the substrate binding pocket. Substitutions at L3 and L5 did not yet turn Epo into a Pla-like invasin, which required more extensive substitutions both at the surface-exposed part of the protein and the membrane-embedded N-terminal region, the β1-strand. Our findings are summarized in Figure 7.

## Methods

### Bacterial strains and plasmids

Bacterial strains and plasmids are listed in Table 1. *pla *and *epo *were available from previous work [19,26]. Derivatives of *pla *and *epo *were cloned by recombinant two-step PCR as described earlier [19,25] and expressed in the inducible pSE380 vector in *E. coli *XL1-Blue MRF as described earlier [19,37]. The nucleotide sequences were confirmed by sequencing. The recombinant *E. coli *strains were cultivated overnight at 37°C in Luria broth (10 ml) supplemented with glucose (0.2% wt/vol), ampicillin (100 μg/ml), and tetracycline (12.5 μg/ml). For protein expression, the culture was pelleted, suspended in 150 μl of phosphate- buffered saline (PBS, pH 7.1), and plated on Luria plates containing 5 μM isopropyl-β-D-thiogalactopyranoside (IPTG) and antibiotics as described above. For the assays, bacteria were collected in PBS, pelleted, and adjusted to an optical density at 600 nm of 1.2 (corresponding to ca. 10^9 ^cells/ml) or 2.0 (2 × 10^9 ^cells/ml).

### Plasminogen activation

Human Glu-plasminogen (4 μg; American Diagnostica), was mixed with 8 × 10^7 ^bacteria in PBS in a total volume of 174 μl in microtiter plate (Nunc) wells, and 30 μl of the chromogenic plasmin substrate Val-Leu-Lys-p-nitroaniline dihydrochloride (S-2251, 2.5 mg/ml; Chromogenix) was added. Plasmin formation at 37°C was measured in a microtiter plate reader at 405 nm.

### Degradation of α_2_-antiplasmin and plasminogen

Cleavage of human α_2_AP (0.8 μg; Calbiochem) was tested as detailed earlier [19,41], by using 3 × 10^7 ^bacteria and the incubation for 2 h at 37°C. Plasminogen degradation with anti-human plasminogen antibody (American Diagnostica) was assessed by incubating 2.5 μg human Glu-plasminogen and 4 × 10^7 ^bacteria in PBS for 2 h shaking at 37°C. The cells were pelleted, and the supernatants were boiled with SDS-PAGE loading buffer. Samples (10 μl) were run in a 12% (wt/vol) SDS-PAGE gel and transferred onto a polyvinylidene difluoride membrane (GE Healthcare). The detection was done with polyclonal anti-human plasminogen antibody (1 mg/ml, diluted 1:1000), peroxidase-conjugated anti-rabbit IgG (1:2500; GE Healthcare), and enhanced chemiluminescence detection reagents (GE Healthcare) according to the manufacturer's instructions. The membrane was exposed to X-ray film (Agfa) for 15 s. In assays where plasminogen cleavage was detected with monoclonal anti-human plasminogen catalytic domain antibody (R&D Systems), the following modifications were made: Glu-plasminogen (3 μg) and 4.8 × 10^7 ^bacteria were incubated for 3 h. After incubation, supernatant samples of 15 μl were electrophoresed and detection was done with the monoclonal antibody (500 μg/ml, diluted 1:500) and peroxidase-conjugated anti-mouse IgG (GE Healthcare; 1:1000). The exposure time was 5 min.

### Degradation of PAI-1/vitronectin complex

Recombinant active human PAI-1 (2.5 μg; American Diagnostica) and human plasma Vn (5 μg; Promega) were incubated for 1 h at 37°C in a volume of 5 μl to form a complex [46]. Bacteria (20 μl, 4 × 10^7 ^cells) in PBS were incubated with the complex for 2 h at 37°C. The bacteria were pelleted, and the supernatants were run in a 12% (wt/vol) SDS-PAGE gel, and PAI-1 and Vn were detected by Western blotting with polyclonal anti-PAI-1 (1:5000, Calbiochem) or anti-Vn (1:1000, Calbiochem) antibodies, alkaline-phosphatase-conjugated anti-rabbit immunoglobulin G (Dako) and phosphatase substrate.

### Detection of Pla-Epo hybrids with anti-Pla, anti-Epo, and anti-Pla loop sera

The cell envelope samples were made by sonicating bacteria (10^9 ^cells/ml) in PBS on ice with 2.5 mM ethylene diamine tetra-acetic acid (EDTA). The remaining cells were pelleted, and the supernatants were centrifuged further. The cell envelopes in the supernatants were pelleted and suspended in PBS and SDS-PAGE loading buffer. Whole-cell samples were made by boiling the bacteria (10^9 ^cells/ml) with SDS-PAGE loading buffer, and the samples were loaded into a 12% (wt/vol) SDS-PAGE gel, electrophoresed, and transferred onto a nitrocellulose membrane. The proteins were detected with a mixture of anti-His_6_-Pla antiserum, antipeptide sera against Pla loops 3 and 5 [19], and anti-His_6_-Epo antiserum (1:1000). The detection was done with alkaline-phosphatase-conjugated anti-rabbit immunoglobulin G (IgG; Dako) and phosphatase substrate.

### Invasion into ECV304 cells

Endothelial-like ECV304 cell line was cultivated in Medium 199 (Gibco) supplemented with 10% fetal calf serum (Gibco) and 2 mM L-glutamine (Gibco) and grown at 37°C with 5% CO_2 _for 3-4 days. Bacterial invasion into ECV304 cells was studied using gentamicin protection assay [47]. ECV304 cells were grown at 24-well plate (Nunc) for 3-4 days. The eukaryotic cells were washed with PBS and Medium 199 was added. Bacteria (10^5 ^cells) in Medium 199 were added, and the plate was centrifuged (128 × g, 10 min). The cells were incubated at 37°C with 5% CO_2 _for 2 h. The wells were washed with PBS, and the extracellular bacteria were killed with gentamicin (100 μg/ml). ECV304 cells were lysed with 0.2% Triton X-100, and the number of intracellular bacteria was determined by viable counting. The invasion percentages were calculated by comparing the amount of the bacteria incubated with the eukaryotic cells to the amount of intracellular bacteria.

### Molecular modelling of protein structures

The omptin structures were modelled on the basis of the crystal structures of Pla [18] (PDB code 2x4m) with program Modeller 9v7 [48] and analyzed visually with program VMD [49].

## Authors' contributions

JH performed the laboratory experiments. LL constructed the homology model and analyzed the structures. BWW helped with the design of the study and writing. TKK supervised the work and mainly wrote the manuscript. All authors participated in planning the work and writing the manuscript and read and approved the final manuscript.
